# On the reliability of non-hermetically encapsulated ceramic printed circuit boards as bridging elements in neural implants

**DOI:** 10.3389/fnins.2026.1911095

**Published:** 2026-08-21

**Authors:** Julien Martens, Lukas Henneken, Alexandra Zehr, Cemre Otyakmazoglu, Paul Čvančara, Thomas Stieglitz

**Affiliations:** 1 Laboratory for Biomedical Microtechnology, IMTEK, University of Freiburg, Freiburg, Germany; 2 BrainLinks-BrainTools Center, IMBIT, University of Freiburg, Freiburg, Germany; 3 Bernstein Center Freiburg, University of Freiburg, Freiburg, Germany; 4 Freiburg Materials Research Center-FMF, University of Freiburg, Freiburg, Germany

**Keywords:** ceramic PCB, neural implants, non-hermetic encapsulation, polyimide, silicone

## Abstract

**Introduction:**

Robust, biocompatible ceramic electrical interconnects are essential for bridging the gap between macro-scale components, such as helically wound leads and connectors, and thin-film polyimide electrodes in neural interfaces. While screen-printed thick-film structures have been utilized for decades, there is a need to broaden the design space and improve reliability through thin-film techniques. This study evaluates various surface configurations to identify the optimal combination of mechanical adhesion, electrical insulation, and functional longevity.

**Methods:**

All samples were fabricated on 96% pure alumina (Al_2_O_3_) substrates. Mechanical stability was evaluated via tensile shear tests, comparing thin-film platinum (Pt) with different adhesion promoters (Si_*x*_N_*y*_, SiO_2_), laser-patterned Pt thin-films, and bare Pt thin-film against reference Al_2_O_3_ substrates. Electrical insulation was assessed by performing electrochemical impedance spectroscopy and DC resistance measurements for degradation monitoring. The evaluated layer stacks with PDMS encapsulation included bare thin-film Pt, thick-film Pt/Au with Overglaze, a SiO_2_ layer, a Si_*x*_N_*y*_ layer, and a layer of pulsed laser deposition (PLD) Al_2_O_3_. All samples underwent accelerated aging in phosphate buffered saline (PBS) at 60°C (acceleration factor ∼ 4.92 vs. 37°C).

**Results:**

Tensile shear strength decreased over the aging period across all groups. However, the integration of adhesion promoters increased mechanical stability compared to bare sputtered Pt, with Si_*x*_N_*y*_ coating bringing adhesion levels close to the Al_2_O_3_ reference samples. Electrically, Si_*x*_N_*y*_-, PLD-Al_2_O_3_, and laser-patterned samples maintained higher insulation impedances over time than PDMS on bare Pt and SiO_2_. DC resistance measurements indicate good capabilities in protecting the conductors from degradation.

**Discussion:**

The findings demonstrate that the addition of adhesion promoters enhances both the longevity of the mechanical bond and stability of the electrical insulation in ion-rich environments. These results provide guidance for selecting optimal ceramic interconnects for chronic peripheral-nerve implants, balancing reliability, manufacturability, and cost.

## 1 Introduction

For most individuals, the execution of daily activities, such as tying shoelaces or opening a bottle, is a seamless process requiring minimal cognitive or physical effort. The loss of one or more limbs resulting from traumatic injury or the onset of paralysis following a stroke or spinal cord injury results in a drastic change in a patient’s lifestyle. Beyond the loss of motor skills and independence, these conditions can also severely restrict the sensory system (Raspopovic et al., 2014). Tactile feedback is essential for perceiving surface properties, enabling the differentiation of object shapes and compliance (Raspopovic et al., 2014), as well as ensuring swift and confident movement on any terrain (Petrini et al., 2019a). Prostheses can provide support by replacing lost limbs and restoring basic motor functionality (Raspopovic et al., 2014; Petrini et al., 2019b). Through advancements in modern medical technology, sensory capabilities can also be regained by stimulating sensory nerve fibers of the peripheral nervous system (PNS) (Raspopovic et al., 2014; Habibollahi et al., 2024).

To achieve this, neural interfaces (e.g., the transverse intrafascicular multichannel electrode (TIME) (Boretius et al., 2010) serve as the link between stimulators, sensorized prostheses, and the PNS (Raspopovic et al., 2014; Petrini et al., 2019b; Clemente et al., 2019). Integrating active tactile and motor feedback significantly enhances the performance of natural activities, including grasping and locomotion, (Raspopovic et al., 2014; Petrini et al., 2019b). The development of minimally invasive neural interfaces takes advantage of established micro-electro-mechanical systems (MEMS) processes, such as spin coating and physical vapor deposition (PVD), which enable miniature cross-sections and high integration densities of stimulation sites (Čvančara et al., 2023; Rubehn et al., 2009). As miniaturization progresses and additional functionalization is integrated, the manufacturability of such devices becomes increasingly challenging due to the added complexity. This is particularly evident when interfacing micro-scale devices with macro-scale components, like connectors, cables, and stimulators. A viable solution is the implementation of a bridging element in the form of an implantable ceramic printed circuit board (PCB). Such an element allows the interfacing electrode to be connected via MEMS-compatible procedures, while the external cable or connector is attached through laser welding or soldering (Kiele et al., 2020). In the specific case of the TIME implant, this bridging element originally consisted of screen-printed conductive metal tracks and contact pads on an alumina substrate (96% Al_2_O_3_), encapsulated by medical grade silicone (Boretius et al., 2012). More recent studies have transitioned these tracks to a thin-film basis (Kiele et al., 2020). This shift toward thin-film fabrication not only enhances precision but also allows for rapid prototyping and quicker design adaptations.

Biocompatibility is a non-negotiable requirement during the fabrication and implantation of medical devices to ensure patient safety. Potentially harmful materials must either be avoided entirely or encapsulated to isolate them from the human body. The implant components themselves must be shielded from bodily fluids to prevent corrosion and ensure long-term stability and functionality. This can be achieved by employing a combination of a stable base substrate and a non-hermetic silicone encapsulation. Kiele showed in Kiele et al. (2020) that alumina substrates and thin-film Pt-based conductors with solder joints are a viable solution for reliable electrical interconnects in the targeted application as a bridging element. By continuing this preliminary work and encapsulating such substrates with medical grade silicone, biocompatibility and mechanical and electrical reliability can potentially be achieved. To enhance the adhesion of the encapsulant different adhesion promoters can be added. The present work is a continuation of Martens (2025), which comprises new layer configurations and additionally to adhesion properties, investigates electrical insulation capabilities of each layer. It focuses on the non-hermetic encapsulation of biocompatible ceramic-based PCBs using alumina (96% Al_2_O_3_) and medical-grade poly-dimethyl siloxane (PDMS) silicone as a base. A conformable, void-free, and long-term reliable coating is essential to prevent water ingress and the subsequent creeping failure of the implanted device. To optimize the interface, different materials are investigated and compared. Finally, accelerated thermal aging in aqueous solution is performed to characterize the degradation behavior of the layers over time (Nanbakhsh et al., 2025).

## 2 Materials and methods

Reliability has been determined by two different kinds of tests, that have been chosen to reflect the expected stress on the material as close as possible to the targeted real-world application. For this purpose, different kind of samples were prepared.

### 2.1 Mechanical testing

By performing tensile shear tests in close analogy to standard DIN EN 1465 the overall adhesion of the encapsulant [NuSil MED 1000, NuSil Technology LLC, Avantor Inc., United States; PDMS (Polydimethyl siloxane)] was investigated. The rationale behind this kind of mechanical adhesion testing lies in the use-case in future applications. As ceramic bridging elements are supposed to connect thin-film electrodes and e.g., cables, the only real expected forces interact in parallel to the substrate’s surface (pulling on the electrode or cable due to movement when implanted in limbs). For this reason, samples were prepared where the investigated surfaces serve as base substrate on which controlled droplets of MED1000 were applied and individual test strips were attached to.

#### 2.1.1 Sample preparation

Samples were separated into five different groups: Alumina substrates with (i) bare sputter deposited platinum (Pt, 500 nm), (ii) Pt coated with 150 nm of silicon oxide (SiO_2_), (iii) Pt coated with 300 nm of silicon nitride (Si_x_N_y_), (iv) laser-patterned Pt, and (v) bare alumina, which served as the control and reference material for subsequent analyses (sample overview in Table 1). The SiO_2_ and Si_x_N_y_ layers were deposited via plasma-enhanced chemical-vapor deposition (PE-CVD). Platinum overall serves as the conducting material on the bridging element. During sputter deposition the entire substrate’s surface is coated, which means a structuring step is required. This can be achieved with laser structuring by selectively removing unwanted material. These exposed areas might show different surface properties compared to pristine alumina samples.

**TABLE 1:** Overview of different sample categories characterized during mechanical and electrical experiments.

Characterization type	Material	Deposition method	Layer thickness
Mechanical characterization	(i) Thin-film Pt	Sputter deposition	500 nm
	(ii) SiO_2_ on thin-film Pt	PE-CVD	150 nm
	(iii) Si_*x*_N_*y*_ on thin-film Pt	PE-CVD	300 nm
	(iv) Laser patterned thin-film Pt	Laser ablation	−
	(v) Al_2_O_3_ (96%)	−	Substrate: 635 μm
Electrical characterization	(a) Thin-film Pt	Sputter deposition	500 nm
	(b) SiO_2_ on thin-film Pt	PE-CVD	150 nm
	(c) Si_*x*_N_*y*_ on thin-film Pt	PE-CVD	300 nm
	(d) Al_2_O_3_ (96%)	Pulsed laser deposition	400 nm
	(e) Pt/Au (5837-G) + Overglaze (4771-P)	Screen printing	20 + 20 μm

**FIGURE 1:**
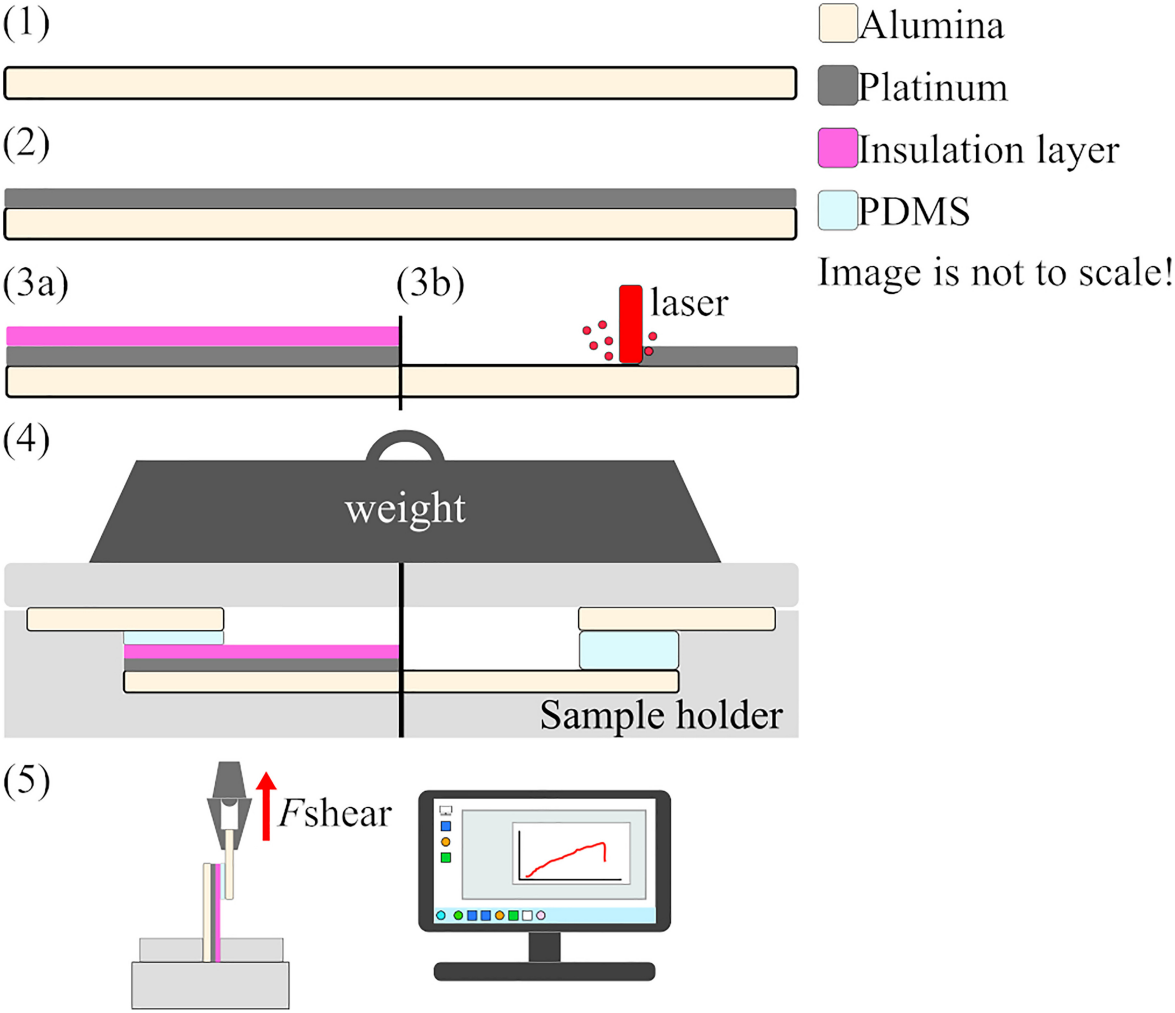
Fabrication process of adhesion test samples. **(1)** Start with clean alumina (96 % Al_2_O_3_) 2“-”ceramic, **(2)** sputter deposition of 50 nm of tungsten-titanium and 500 nm of platinum (Pt), **(3a)** deposition of adhesion promoters (SiO_2_, Si_*x*_Ny,) or **(3a)** laser removal of metal layer for re-exposed alumina, **(4)** application of defined amount of silicone (MED 1000) to sample and adding a 25 mm x 2 mm alumina strip for pulling. With the help of a self-made sample holder the gap between the ceramic and alumina strip was kept at 100 μm and the overlapping area was defined as 2 × 5 mm. A 5 kg weight was added during curing to firmly and evenly press on the samples, **(5)** pull shear tests performed with a multipurpose bond tester, recording of force graphs.

Two-inch (50.8 × 50.8 mm, 630 μm in thickness) alumina (Al_2_O_3_) ceramic substrates (Rubalit, 96 %, A.L.L. Lasertechnik GmbH, Munich, Germany) were first cleaned for 1 min in a stirred “Leslie’s Soup” mixture (0.5 wt% Teepol-L detergent (Teepol Products, Kent, United Kingdom), 2.5 wt% sodium phosphate (Na_3_PO_4_), 97 wt% de-ionized water) and then for an additional minute in agitated isopropanol (Kiele et al., 2021). The substrates were rinsed and subjected to a 10-min ultrasonic bath in de-ionized water. Drying was carried out with a pressurized stream of nitrogen (N_2_). The cleaned substrates were subsequently moved into an ISO-4 cleanroom for the upcoming metallization step. There they underwent a second cleaning sequence: alternating acetone and isopropanol baths with ultrasonic assistance, followed by a rapid rinse in de-ionized water. Finally, the ceramics were placed on a hot plate to dry at 110°C for 15 min (Figure 1(1)).

Metallization was performed by sputter deposition (FHR star 100–PentaCo, FHR Anlagenbau GmbH, Ottendorf-Okrilla, Germany) of a 50 nm tungsten-titanium (WTi) adhesion layer, immediately followed by 500 nm of platinum (Pt), as described in (Kiele et al., 2020) for robust conductors in active implants (Figure 1(2)). Samples of the groups (ii) and (iii) were introduced into a PE-CVD machine (STS Surface Technology Systems GmbH, Newport, United Kingdom) for 150 nm of SiO_2_ and 300 nm of Si_*x*_N_*y*_ deposition (Figure 1(3a)). The laser-patterned Pt samples, were fabricated with a nanosecond pulsed laser (Nd:YAG, DPL Genesis Marker, ACI Laser-Components, Nohra, Germany), where patches with a size of 6 mm × 3 mm were cleared from the metal layer to reveal the underlying Al_2_O_3_ (Figure 1(3b)).

The coated substrates were positioned in a purpose-built sample holder (Figure 1(4)). MED 1000 was dispensed onto the substrate using a dispensing unit (dispensing time = 9 s, pressure = 3 bar, nozzle = 0.5 mm). Laser-cut Al_2_O_3_ test strips (2 × 25 mm^2^, cleaned in isopropyl alcohol and DI-water for 10 min each, in ultrasonic bath, dried under nitrogen stream) were then laid atop the PDMS, producing a defined overlap area of *A*_*overlap*_ = 2 × 5 mm^2^ as ensured by the holder. The gap between PDMS and the strip was set to a constant 100μm. The assemblies were left to cure for 72 h. Once fully dried, each substrate was halved to facilitate handling during subsequent testing (Figure 1(5)).

#### 2.1.2 Adhesion characterization

All samples were sterilized in a calibrated tabletop autoclave (Superior B23, Mammooth srl, Bibbiano, Italy) in a standardized process (DIN EN ISO 17665-1) for 19 min at 121°C and 1.06 bar. After sterilization, they underwent accelerated thermal aging in phosphate-buffered saline (PBS) at 60°C for periods of up to 70 days. Incorporating a sterilization step into the manufacturing flow brings the test conditions closer to those encountered in actual practice, since any device destined for human use must be sterilized before implantation. Accelerated thermal aging mimics the long-term mechanical and chemical stresses that materials experience during their intended use by exposing them to elevated temperatures for shorter times; the degradation can then be extrapolated to roughly predict life-time under normal conditions. The idea is that, under the assumption of the Arrhenius equation, the reaction rates of ongoing processes increase due to the higher heat input, and thus potential failure mechanisms emerge earlier (hence “accelerated aging”). In literature, a highly simplified rule is often assumed: reaction rates double for every 10 K increase in temperature (Hemmerich, 1998). This leads to Formula (1), which can be used to calculate an acceleration factor *a*. However, this factor should be treated with caution and does not necessarily reflect reality. Two simplifying assumptions are inherently made to calculate the acceleration factor: a) the dominant failure mechanism does not change with higher temperature and b) the environmental conditions next to temperature and the activation energy of the investigated failure mechanism remain unchanged (Shah et al., 2026). Even small deviations can lead to drastically different or incorrect results, leading to a drastic underestimation or in the worst-case overestimation of the implant’s actual lifetime. Emerging the samples in PBS tries to replicate physiological conditions by offering high relative humidity and a saline surrounding. Within these limitations, an acceleration factor of *a* ≈ 4.92, derived from Equation (1) with *T*_1_ = 60°C and *T*_*ref*_ = 37°C (physiological temperature) can be assumed. The 70-day exposure at 60°C then corresponds to roughly 345 days at body temperature (Hukins et al., 2008; Hemmerich, 1998).


$$a=2^{\frac{T_{1}-T_{\mathrm{ref}}}{10}}$$ (1)

Tensile-shear testing was employed to quantify the adhesion of PDMS to its underlying substrates. The measurements were carried out on a calibrated multipurpose bond tester (Cartridge WP10KG; Dage4000, Nordson GmbH, Erkrath, Germany). Each specimen was secured in the device and the test strips were pulled at a constant rate of 100 μm s^−1^. The instrument recorded the peak tensile-shear load*F*_*shear*_ required to cause failure (Figure 1(5)). The corresponding tensile-shear strength τ was then obtained from *F*_*shear*_ and the defined overlap area *A*_*overlap*_ = 10 mm^2^ according to Equation (2).


$$\tau =\frac{F_{\mathrm{shear}}}{A_{\mathrm{overlap}}}$$ (2)

#### 2.1.3 Optical analysis

To confirm that Equation (2) can be applied, the specimens were inspected optically after the shear test. The prerequisite is that the fracture be adhesive (separation at material interface), rather than cohesive (break within material itself) and thus leave the overlapping area *A*_*overlap*_ undefined. Before sample assembly and after the tensile shear tests, SEM images (Helios 5 CX ThermoFisher Scientific Inc., United States) were taken of the surfaces to acquire their morphology as well as potential changes at the material interface.

#### 2.1.4 Statistical analysis

For statistical analysis, a linear model to describe the decay of the adhesion over time and estimate the influence of the layer combination and time of aging was used, as well as their interaction. A linear model with robust standard errors was used to account for any observed heteroscedasticity. A double tailed *t*-test was performed on the dataset with the reference sample group Al_2_O_3_ to test for significance. The initial adhesion at zero days of aging ( = “intercept”) as well as the influence of the duration of aging ( = “time”) was compared.

To confirm the usability of such a model, diagnostic plots were evaluated and a Shapiro-Wilk test was performed to investigate whether the residuals are normally distributed ( = null hypothesis H_0_). If so, the model is assumed to be adequate. The significance level α is set for all analysis to 0.05, everything computed in R version 4.5.3 using packages readxl to import excel files, ggplot for figures, and sandwich for heteroscedasticity-consistent covariances (HC3).

### 2.2 Electrical insulation testing

#### 2.2.1 Sample preparation

For electrical insulation testing, the samples were categorized into five groups: (a) bare Pt (reference), (b) SiO_2_, (c) Si_x_N_y_, (d) pulsed laser deposition (PLD) Al_2_O_3_, and (e) thick-film Pt/Au (5837-G Pt/Au conductor, Vibrantz GmbH, Germany) + overglaze (GS OVERGLAZE 4771-P, Vibrantz GmbH, Germany) (sample overview in Table 1). Groups (a) through (d) utilized the same thin-film Pt as detailed in section 2.1.1, prepared according to the identical protocol. Prior to the application of any adhesion-promoting layers, the samples were structured using a nanosecond pulsed laser (DPL Genesis Marker, ACI Laser-Components, Nohra, Germany). The chosen design reflects track dimensions from an established process in our laboratory. Ceramic PCBs fabricated via screen printing have demonstrated mechanical and electrical robustness and reliability in our testing (Schuettler et al., 2010) and across several human trials for up to 6 months (Čvančara et al., 2023; Petrini et al., 2019a).

Two distinct sample geometries were designed: (1) A configuration comprising three parallel conductive tracks, each 11.2 mm in length with a width of 180 μm and a center-to-center pitch of 250 μm. These were used for electrochemical impedance spectroscopy (EIS) to characterize the insulation properties between the encapsulated tracks and the surrounding conductive medium (PBS, Sigma Aldrich, United States). (2) A meander structure with the same track width and a total path length of 45 mm between contact pads. These were employed for DC resistance measurements to monitor the degradation or structural changes of the overall layer stack.

Adhesion promoting layers serving as insulation layers simultaneously were added by different means: SiO_2_ (150 nm) and Si_*x*_N_*y*_ (300 nm) were both deposited by PE-CVD, pulsed laser deposition (PLD) for PLD-Al_2_O_3_ (400 nm, process pressure = 5 × 10^–6^ mbar, frequency = 10 Hz, wavelength = 248 nm, 40,000 shots), and screen printing for the thick-film devices (layer height of PtAu paste: 20 μm, Overglaze: 2 × 10μm).

**FIGURE 2:**
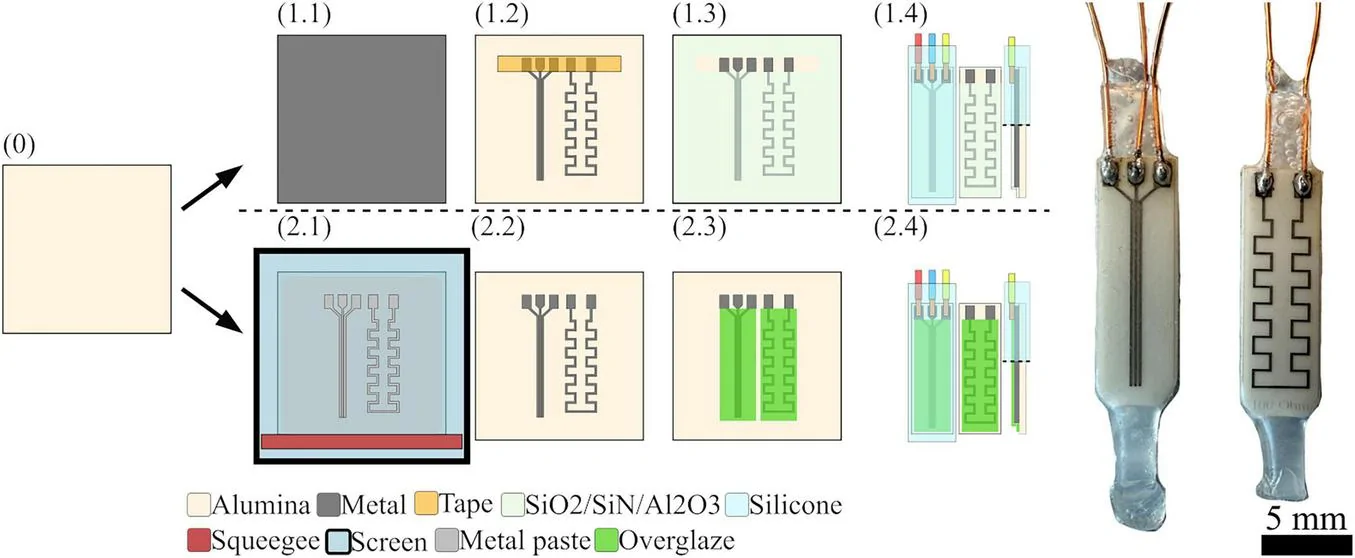
Device assembly procedure for insulation impedance and DC resistance measurements. (0) Start with clean alumina (96 % Al_2_O_3_) 2“-”ceramic. (1) describes the procedure for thin-film samples: (1.1) metallization with 50 nm of tungsten-titanium and 500 nm platinum (Pt), (1.2) laser-patterning of metallized ceramics and masking the solder pads with Kapton^®^ tape, (1.3) deposition of adhesion promoter/insulation layer (SiO_2_, Si_*x*_N_*y*_, PLD alumina) and removal of tape to expose the solder pads, (1.4) soldering of copper cables to samples, adding the silicone tubing (MED 4750, 0.2 mm thickness) and filling out the tube with silicone (MED 1000); (2) describes the procedure for screen-printed samples: (2.1) screen-printing of PtAu-paste on ceramic, (2.2) firing in the metal paste under oxygen atmosphere, (2.3) screen-printing of overglaze and burning in as insulation layer, repeated two times, (2.4) soldering of copper cables to samples, adding the silicone tubing (MED 4750, 0.2 mm thickness) and filling out the tube with silicone (MED 1000). Photo shows real samples for insulation impedance measurements (left) and DC resistance measurements (right). The left sample provides three equal length parallel conductors that can be tested for electrical insulation in a pair-wise configuration. The right sample consists of an equal trackwidth meander structure.

The final samples showed dimensions of 4.5 × 15 mm × 630 μm (W × H × D), with copper wire (diameter 0.28 mm, length 50 mm) soldered for electrical connection. To encapsulate the devices, medical-grade silicone tubing (NuSil MED 4750, 2 mm ID, 2.6 mm OD, 30 mm length) was swelled in petroleum ether to allow it to be pulled over the samples. Immediately following this, the tubing was filled from the bottom side with MED 1000 via a dispensing system (3–4 bar, nozzle diameter 0.5 mm) until the silicone emerged from the opposite end, covering the contact pads and beyond. This is according to the same procedure as during the TIME device assembly. The samples were left to cure for 3 days and subsequently sterilized in an autoclave at 121°C and 1.02 bar for 19 min. The fabrication flow-chart and design are illustrated in Figure 2.

Group (e) was fabricated using a screen-printing process. First, PtAu paste (5837-G, Vibrantz GmbH, Germany) was printed onto the ceramic substrate and fired according to the manufacturer’s specifications. Subsequently, an overglaze (4771-P, Vibrantz GmbH, Germany) was applied to provide electrical insulation for the PtAu tracks and was similarly fired. The overglaze was applied twice to ensure a pinhole free insulation layer. The overall dimensions and the encapsulation procedure remained identical to those described previously.

#### 2.2.2 Insulation characterization

**FIGURE 3:**
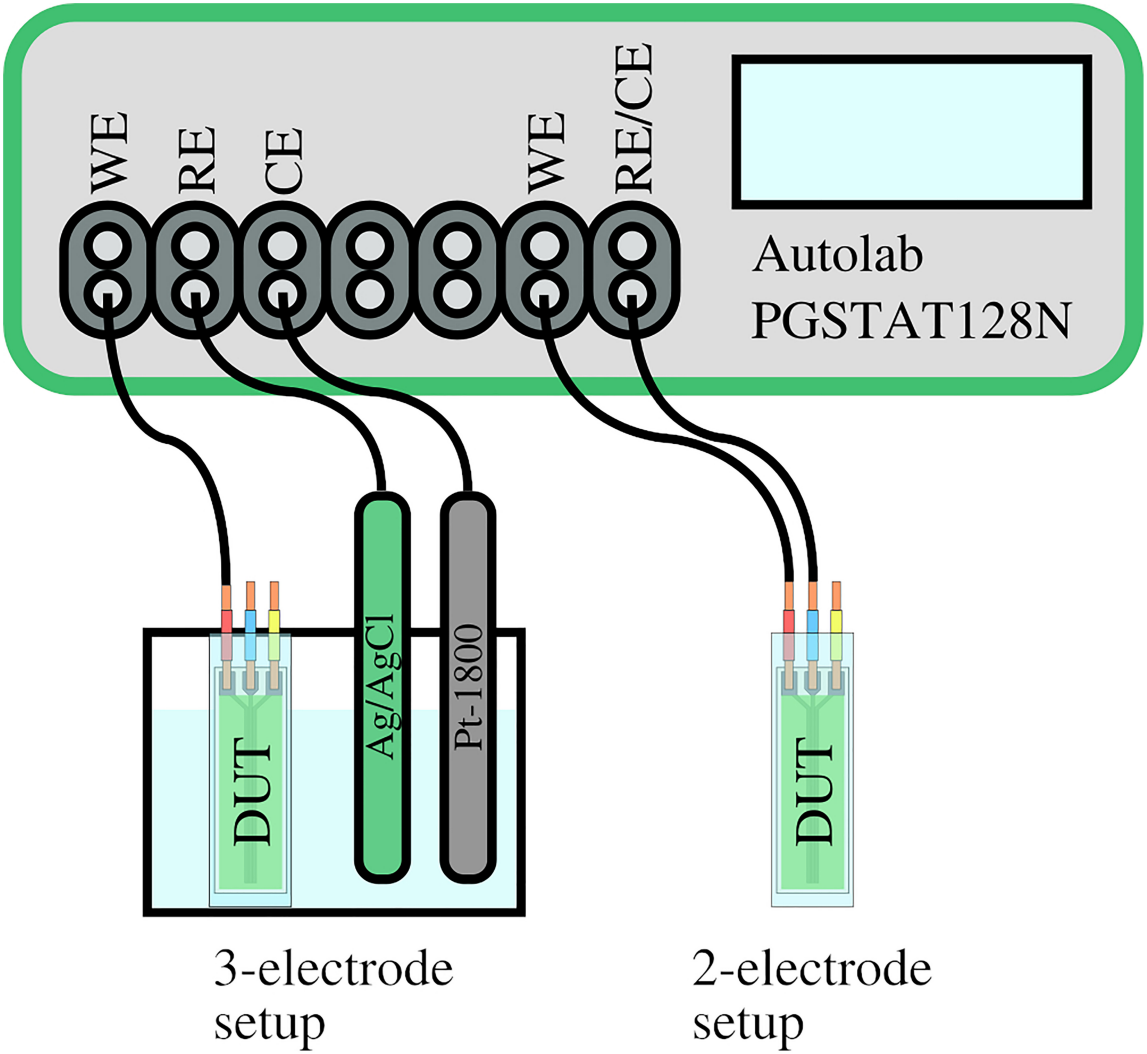
Setup for insulation impedance measurements. Left: The device under test (DUT), the silver/silver-chloride reference (RE), and platinum counter electrode (CE) are submerged in PBS and connected to the potentiostat (3-electrode setup). Individual channels [ = working electrode (WE)] are measured via electrochemical impedance spectroscopy (EIS) toward the surrounding media. Right: The DUT is attached to the potentiostat. Either channel 1 and 2 or channel 2 and 3 are connected as WE and RE/CE (2-electrode setup) and an EIS measurement is conducted to collect data on insulation impedance and phase.

Samples were initially characterized immediately following manufacturing before accelerated aging (dry vs. wet states). Sample type 1) was connected to a potentiostat (PGSTAT128N, Metrohm Autolab B.V., Utrecht, The Netherlands) in two configurations: First, in a three-electrode setup (Figure 3) in order to measure the insulation capabilities of the encapsulant toward the surrounding PBS media of the center channel, which served as the working electrode. A Pt-1800 (SI Analytics GmbH, Mainz, Germany) served as the counter-electrode (CE), while a 3M KCl silver/silver-chloride (Ag/AgCl) electrode (SI Analytics GmbH, Mainz, Germany) was used as the reference electrode. A sinusoidal signal with an amplitude of 10 mV was applied across a frequency range of 100 kHz to 1 Hz, and the resulting impedance and phase were extracted at 1 kHz.

Second, the setup was changed to a two-electrode setup to identify the insulation impedance between neighboring tracks. Two pair configurations were possible: Channel 1 (WE) and Channel 2 (RE/CE), and Channel 2 and 3 as WE and RE/CE, respectively. Samples were taken out of the PBS. The same settings as before were chosen to perform these measurements. Again, phase and impedance were extracted at 1 kHz. After initial measurements, the samples were placed in an air-tight container with PBS, being submerged up until the cables, at 60°C in an incubator and aged for up to 4 weeks. Measurements were taken weekly.

#### 2.2.3 DC resistance measurements for degradation characterization

Samples of type 2) were measured with a multimeter (HP 34401A, HP Inc., United States) in a 2-point wire configuration and the DC resistance of the Pt meander structure was acquired. Afterward the samples were inspected under a light microscope to observe any visual degradation signs that might be linked to resistance change. After initial measurements, the samples were aged according to the previous protocol as well. Measurements were taken weekly.

## 3 Results

### 3.1 Mechanical characterization

#### 3.1.1 Overall observations

**FIGURE 4:**
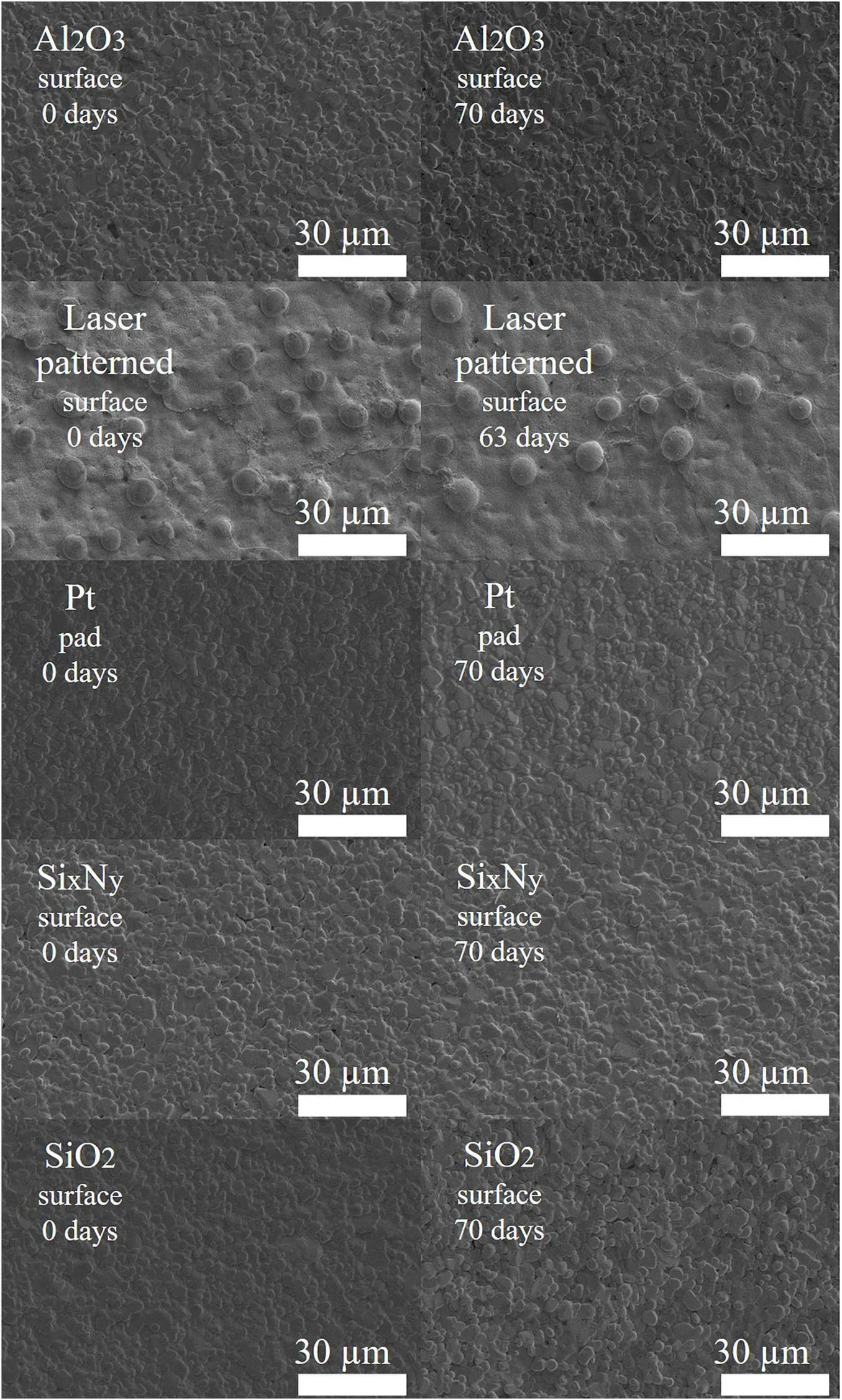
Scanning electron microscope (SEM) images of tested surfaces. No significant changes could be observed over time of aging for most groups. SiO_2_ shows different morphology on surface areas that were exposed toward the saline solution. Areas that were covered by the test samples showed no difference.

Optical inspection with the help of a microscope confirmed that the silicone patch covered an area of 2 × 5 mm^2^. SEM images shown in Figure 4 reveal no significant changes in the surface morphology before and after aging for most of the samples. As only Pt and SiO_2_ samples showed adhesive failure on the sample surface side, it was only possible to acquire imaging data from these test sites. Any try in post-testing removal of the remaining silicone resulted in altering the surface thus not representing the morphology only influenced by the tests themselves. Therefore, the overall influence of aging in saline solution of the different surfaces was monitored. SiO_2_ showed no change in surface morphology under the silicone, but the remaining exposed surface noticeably changed in texture. For all other groups, no changes could be observed.

**FIGURE 5:**
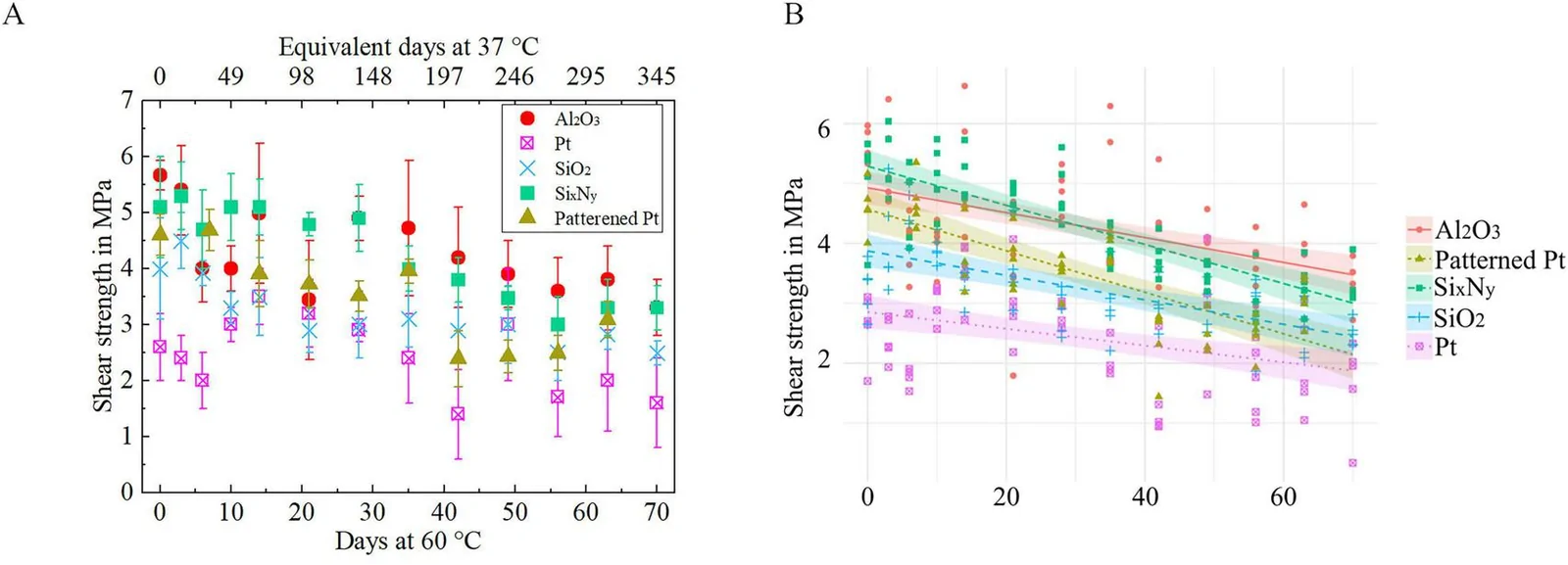
**(A)** Graph showing the adhesion decay over time of aging at 60°C in phosphate buffered saline (PBS) (*n* = 5). Trend for all is a lower adhesion over time. Alumina shows highest adhesion overall while platinum (Pt) without adhesion promotion shows lowest. The top *x*-axis shows the equivalent days at 37°C body temperature. **(B)** Bonding strength (MPa) as a function of time (days) for various surface treatments [Al_2_O_3_, laser-structured, Si_*x*_N_*y*_, SiO_2_, and platinum (Pt)] during aging. Solid lines represent the linear regression of the mean, with shaded areas indicating the 95% confidence intervals. Individual data points represent individual sample measurements.

The mechanical characterization of the samples (for each group *n* = 5) revealed progressive degradation over time of aging for all groups (Figure 5). Failure mechanisms showed a varying behavior, although all being of adhesive nature (meaning the silicone fully detached from the interface). While the samples of the groups Si_*x*_N_*y*_, Al_2_O_3_, and laser-patterned Pt always showed silicone detachment from the test strips, the samples with bare Pt lost adhesion from the samples’ surfaces, revealing darker areas underneath the silicone. The samples with SiO_2_ layers changed the location of adhesive failure. For the first week failure was exclusively observed at the interface with the test strips. During testing after 10 and 14 days, silicone detached two times from the test strips and three times from the samples’ surfaces, also revealing darker patches underneath. From 3 weeks of aging on, failure happened exclusively on the SiO_2_ surface.

Bare Al_2_O_3_ exhibited the highest overall adhesion, decreasing from 5.7 ± 0.3 MPa to 3.3 ± 0.5 MPa. Si_*x*_N_*y*_ followed a similar trend, starting at 5.1 ± 0.9 MPa and ending at 3.3 ± 0.4 MPa. SiO_2_ showed lower initial and final values (4.0 ± 0.9 MPa and 2.5 ± 0.2 MPa, respectively), while Pt demonstrated the weakest performance, dropping from 2.6 ± 0.6 MPa to 1.6 ± 0.8 MPa after 70 days of aging (Figure 5).

#### 3.1.2 Statistical analysis

**FIGURE 6:**
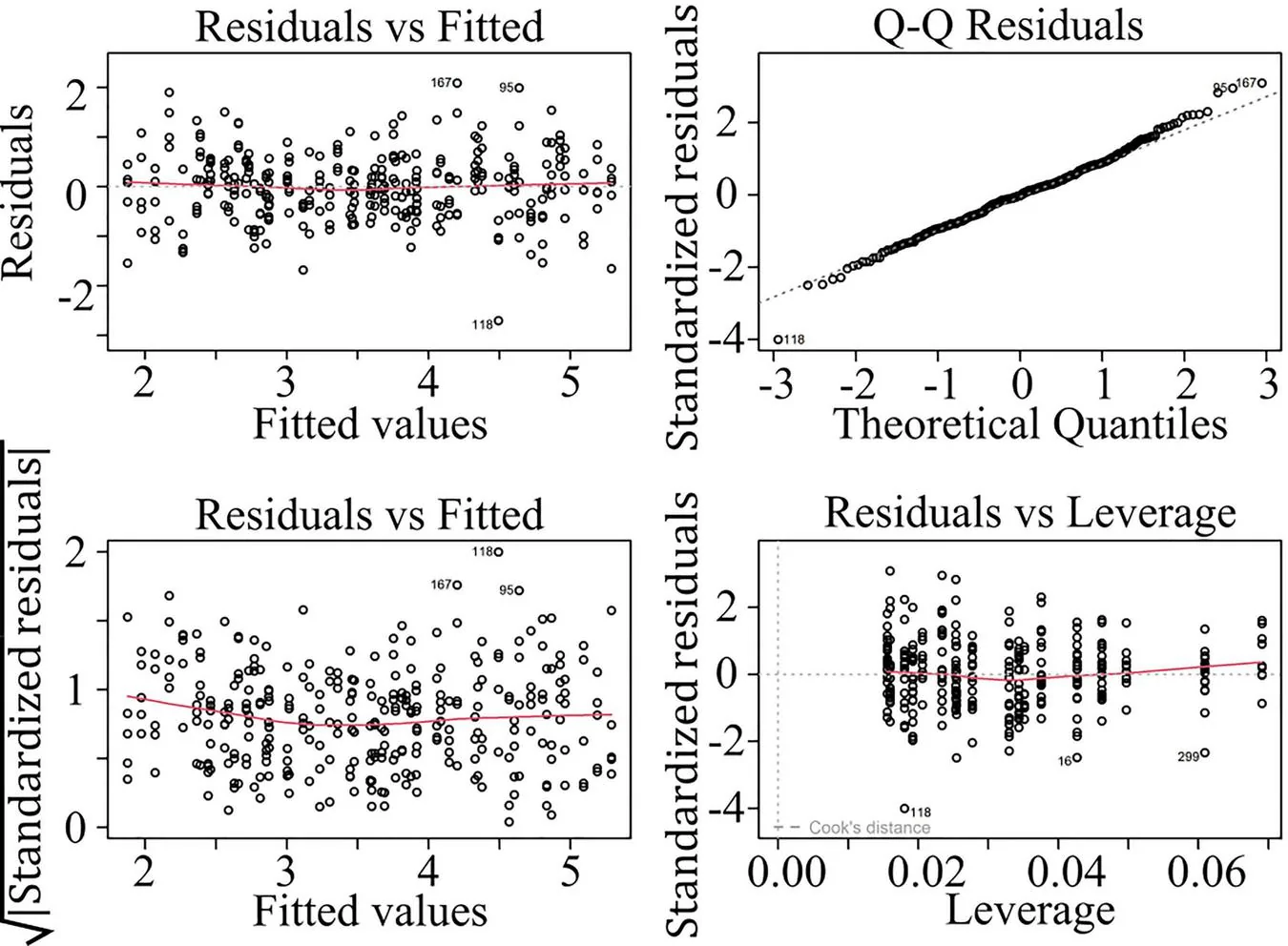
Diagnostic plots for linear model analysis. Residual vs. Fitted: An equal spread of residuals around the horizontal line indicates linear relationship in the model. Q-Q Residuals: Linearity of the residuals indicates normal distribution of the data. Scale-Location: Random distribution of residuals rejects heteroscedasticity. Residuals vs. Leverage: Residual distribution well within Cook’s distance (not visible in plot as it is too large) shows no outliers.

The Shapiro-Wilk test of the data after applying the linear model computed a *p*-value of 0.1202 (>0.05). This means that H_0_ can be accepted and H_1_ rejected; there is sufficient evidence for normally distributed residuals, which means that the assumption that they are normally distributed can be taken. Results from the diagnostic plots (Figure 6) indicate that the linear model of the dataset can be employed for further analysis.

*T*-test results of the data of the Al_2_O_3_ group shows strong significance (p_*Al*2O3, *intercept*_ = < 2.2 × 10^–16^, p_*Al*2O3, *time*_ = 1.79 × 10^–7^) with p_*Al*2O3_ < < 0.05. The *t*-test for the remaining groups (laser-patterned, Si_*x*_N_*y*_, SiO_2_, Pt) compared to the reference Al_2_O_3_ shows varying significance levels. Laser-patterned surfaces as well as Si_*x*_N_*y*_ show no significant difference in initial adhesion (p_*laser*–*patterned*,intercept_, p_*SixNy*, *intercept*_ > 0.05) but significant stronger decline in adhesion over time (p_*laser*–*patterned*,time_, p_*SixNy*, *time*_ < 0.05). Exact t values and *p*-values can be found in Table 2. For SiO_2_ and Pt samples the opposite occurred. While their *p*-values for the intercept showed strong significance, time seems to not have a different effect compared to Al_2_O_3_.

**TABLE 2:** Summary of the second linear model with heteroscedasticity-robust standard errors.

Material	Parameters	Estimate	Std. error	*T*-value	*P*-value
**Al_2_O_3_**	Intercept	4.928	0.192	25.690	**< 2.2e-16**
	Time	−0.0208	0.0039	− 5.325	**1.79e-07**
**Laser-patterned**	Intercept	−0.359	0.236	−1.524	0.1286
	Time	−0.0139	0.0057	−2.422	**0.0160**
**Si_*x*_N_*y*_**	Intercept	0.361	0.229	1.573	0.1168
	Time	−0.0119	0.0049	−2.428	**0.0158**
**SiO_2_**	Intercept	−1.054	0.236	−4.465	**1.14e-05**
	Time	0.0003	0.0049	0.066	0.9478
**Pt**	Intercept	−2.074	0.241	−8.609	**4.23e-16**
	Time	0.0068	0.0057	1.185	0.2368

The units for the intercept are MPa, and for time MPa/day. Highlighted *p*-values confirm H_0._

### 3.2 Electrical performance

#### 3.2.1 Insulation toward surrounding media

**FIGURE 7:**
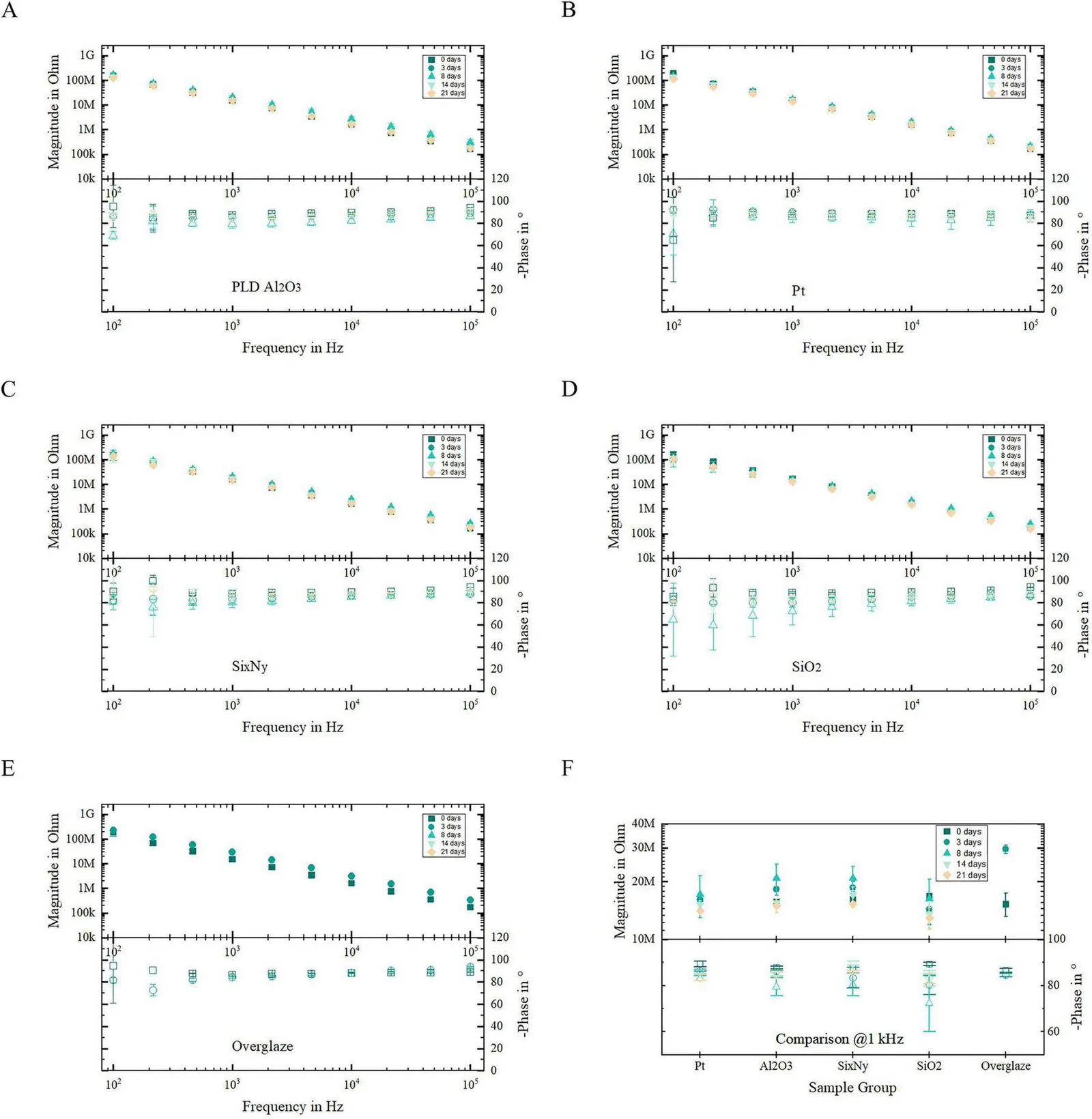
Insulation impedance of all sample groups toward surrounding medium (each *n* = 3). The panels show the different investigated materials insulation layers: **(A)** PLD Al_2_O_3_, **(B)** bare Platinum (Pt), **(C)** SixNy, **(D)** SiO_2_, and **(E)** screen printed overglaze. **(F)** Allows for more detailed comparison of impedance andphase at 1 kHz. All layer types show similar insulation capabilities up to 3 weeks of aging. SiO_2_ shows widest variation in phase and impedance.

Over the observed time frame for each individual group no significant change in impedance was observed (Figure 7). For better comparison, each value at 1 kHz was extracted and faced against each other. Overall, the impedance values and phases are in the same shallow range, with similar changes over time. Initial impedances hint toward minor differences in the ability of electrical insulation.

#### 3.2.2 Insulation between neighboring conductor tracks

**FIGURE 8:**
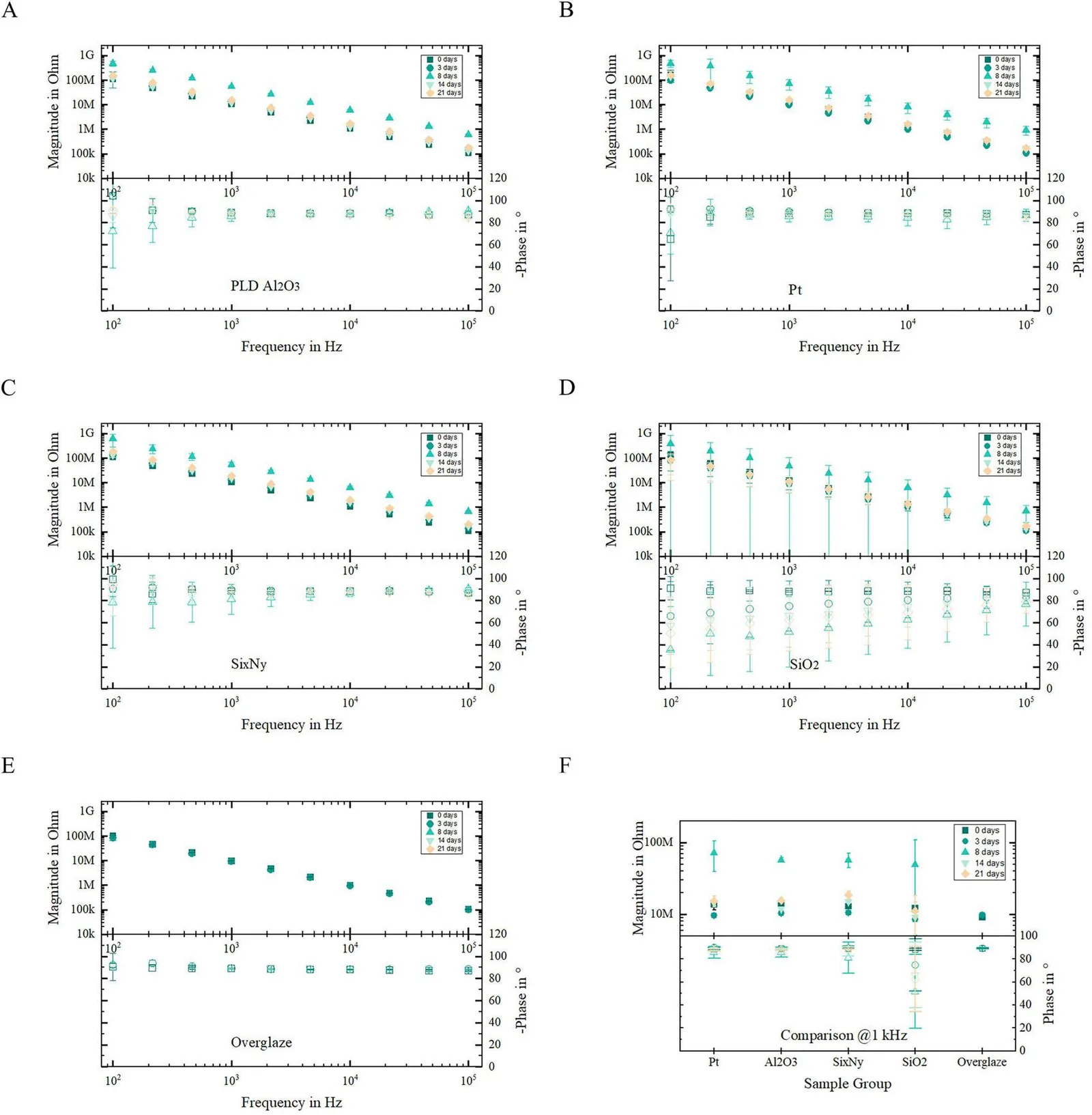
Insulation impedance of all sample groups between neighboring conductor tracks (each *n* = 6). Samples have been provided with different insulation layers and are represented in the panels as **(A)** PLD Al_2_O_3_, **(B)** bare Platinum (Pt), **(C)** SixNy, **(D)** SiO_2_, and **(E)** screen printed overglaze. **(F)** gives detailed comparison of impedance and phase at 1 kHz. Similar insulation capabilities can be seen up to 3 weeks of aging. SiO_2_ shows wide changes in phase and impedance.

No significant change in impedance of each individual group was observed (Figure 8). For better comparison, each value was also extracted at 1 kHz and plotted together. Overall, the impedance values and phases are in the same range, with similar changes over time. The jump in impedance on the eighth day for the sample groups (a) Pt, (b) SiO_2_, (c) Si_*x*_N_*y*_, and (d) PLD-Al_2_O_3_ can be attributed to a systematic error and is further discussed in section 4.2.1. It can be observed that SiO_2_ samples evoke a noticeable change in phase after 3 days of aging. The other sample groups show smaller changes in phase.

#### 3.2.3 Encapsulated Pt meander DC-resistance

**FIGURE 9:**
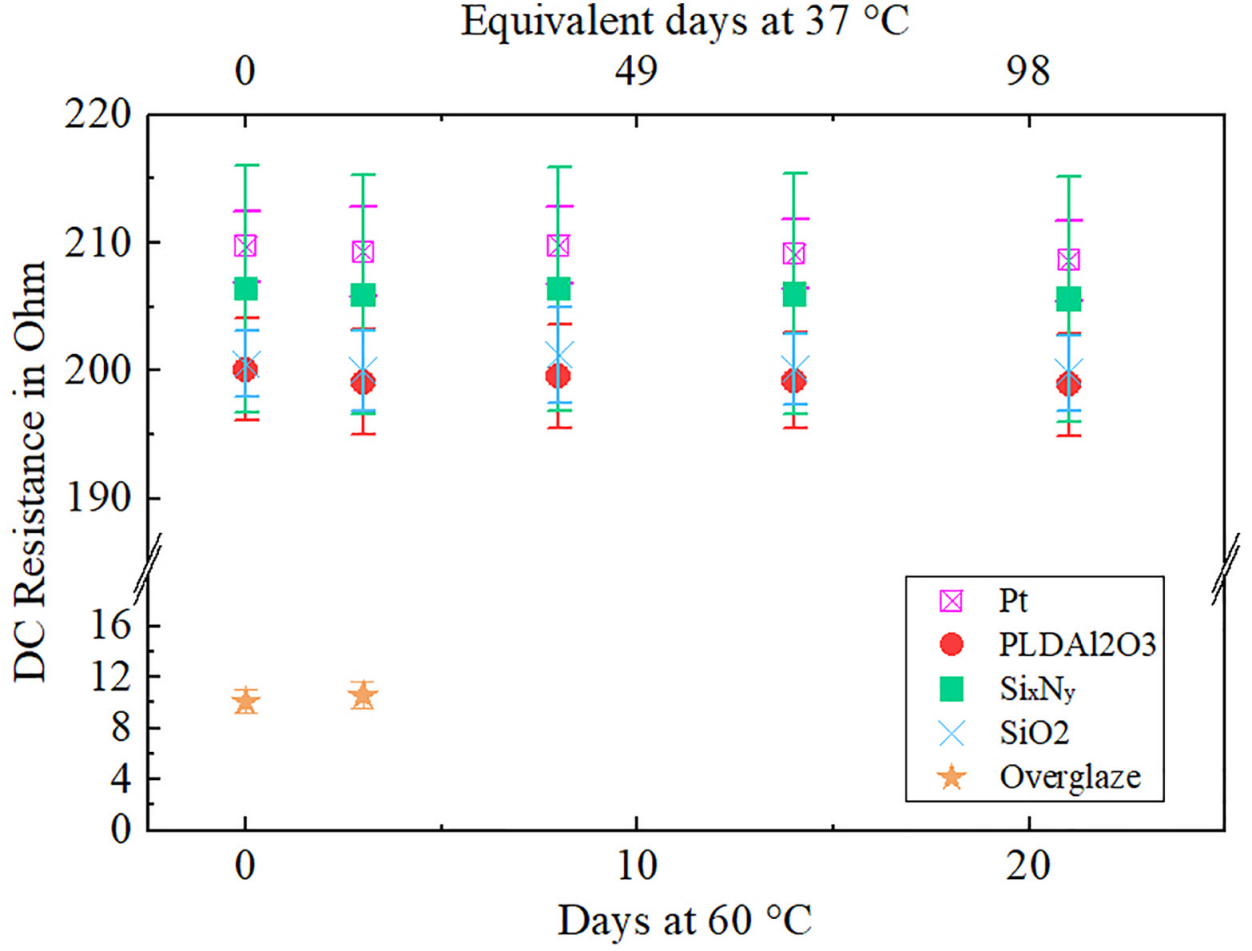
DC resistance measurements (***n*** = 3 for each data point). Overall stability of the resistance values across all groups has been observed. Lower resistance values of Overglaze samples compared to all other are due to larger conductor cross-section and different material (PtAu vs. Pt).

After repeated measurements in the time frame of aging, DC resistance showed small changes for all groups (Figure 9). While the thin-film samples’ DC resistance remained similar over the observed time of aging, the thick-film (Overglaze) samples increased in resistance over the first days. Overall electrical resistance is lower for the thick-film samples compared to the thin-film ones. Platinum samples show the highest resistance on average, while samples covered with PLD-Al_2_O_3_ or SiO_2_ showed lowest.

## 4 Discussion

### 4.1 Mechanical performance and morphology

Surface treatments significantly influenced the adhesive strength and durability of the tested materials. All samples exhibited a consistent decline in adhesion during aging, likely driven by water uptake within the silicone matrix. PDMS is known to swell upon water absorption, inducing mechanical stresses at the interface that counteract adhesive forces (Khan et al., 2018). Furthermore, the infiltration of water into available micro-cavities or -voids may have disrupted interfacial contact or accelerated the propagation of interfacial failure (Nanbakhsh et al., 2025).

The mechanical degradation varied by surface treatment and morphology. Al_2_O_3_ and Si_*x*_N_*y*_ exhibited the highest initial adhesion (5.7 ± 0.3 MPa and 5.1 ± 0.9 MPa, respectively). The laser-patterned group also showed no significant difference in initial adhesion compared to the Al_2_O_3_ reference. However, both Si_*x*_N_*y*_ and the laser-patterned surfaces exhibited a significantly stronger decline in adhesion over time (*p* < 0.05 compared to the reference). This suggests that while surface patterning and specific coatings can maximize initial bond strength, they may not provide the same long-term stability as the Al_2_O_3_ reference, potentially due to an increased surface area and minor residues that facilitates water accumulation at the interface.

In contrast, SiO_2_ and Pt demonstrated significantly lower initial adhesion (*p* < 0.05), yet their rate of decline was not statistically different from the Al_2_O_3_ reference. This indicates a lower interfacial energy. A critical observation was the shift in failure mechanisms for SiO_2_; while failure occurred at the test-strip interface during the first week, it shifted exclusively to the SiO_2_ surface after 14 days. This transition indicates that the SiO_2_-PDMS interface became the weakest combination as aging progressed. For bare Pt, the failure was consistently localized at the sample surface, confirming it as the weakest overall interface.

Adding Si_*x*_N_*y*_ as adhesion promoter to Pt-based samples achieved durability and adhesion levels comparable to bare Al_2_O_3_. This demonstrates that the optimization of adhesion-promoting interlayers is a viable pathway for enhancing long-term device longevity.

To ensure the measured values reflected interfacial adhesion rather than the cohesive properties of the PDMS, the silicone layer was kept intentionally thin. A thicker coating would have increased the cross-sectional area and allowed for greater deformation, reducing overall stiffness and biasing the results toward the cohesive strength of the bulk silicone. While these results provide preliminary indications of material behavior, they must be interpreted with caution. Accelerated aging at elevated temperatures may introduce thermal effects and degradation mechanisms that are absent under physiological conditions at 37°C. Since the underlying activation energy in the Arrhenius equation is hard to estimate, small variations already lead to large changes due to the exponential behavior of this change.

### 4.2 Electrical insulation stability

#### 4.2.1 Insulation impedance

The electrochemical impedance spectroscopy results demonstrate that the PDMS encapsulation provides a stable electrical insulation barrier across all tested surfaces, with no significant degradation in insulation performance over the observed aging period. The consistency of the impedance values and phases at 1 kHz suggests that the dielectric properties of the PDMS remained largely unaffected by the underlying substrate.

The similarity of impedance values across all groups indicates that the bulk insulation properties of the silicone dominate the electrical response, rather than interfacial interactions. However, a notable divergence was observed in the SiO_2_ group, which exhibited a noticeable phase shift after 3 days of aging. Given that the impedance magnitude remained stable, this phase shift likely indicates a change in the capacitive behavior at the SiO_2_-PDMS interface, possibly related to the preferential accumulation of water molecules or ions at this specific interface. In the case of accumulation of water molecules at the interface, if any cavities are available, water can condensate and lead to SiO_2_ dissolution (Kang et al., 2014) and open up tiny resistive pathways, that are invisible under a light microscope until now. Resulting delamination at the microscale can further open up pathways and cause system failure. If these small resistive pathways are available, they could be modeled as a high-resistance component parallel to a capacitor, dominating the impedance behavior as can be seen in Figure 8D. This would be in line with the drop in impedance from 12.3 MOhm on day zero to 8.5 MOhm on day 3, with a phase shift from 88 to 75° at 1 kHz, dropping further down to ∼60° as the experiment continues. Capacitive behavior is still dominating but the continuous decrease in phase hints toward growing resistive behavior and thus higher water accumulation at the interface due to delamination or SiO_2_ breakdown ( = more resistive pathways). This finding correlates with the observed shift in adhesive failure location for the SiO_2_ group.

Regarding the data processing, measurements below 100 Hz were excluded to remove resolution-switching artifacts inherent to the potentiostat’s settings, ensuring that only high-fidelity data were analyzed. Furthermore, the synchronized impedance spike observed on day eight across all samples was identified as a systematic measurement error. During all measurements that day, the electrical contact to the measuring device was poor and caused a shift by an order of magnitude in the measurement data. The reason why sample group (e) Overglaze did not express any sudden jump in impedance on the eighth day of accelerated aging is attributed to the fact that these measurements have not been performed on the same calendar day as with the remaining groups. Because the values returned to the previous baseline immediately following this event, this data point was treated as an outlier and does not reflect a material-driven phenomenon.

#### 4.2.2 DC-resistance

The DC resistance measurements of the platinum meander structures were conducted to evaluate the effectiveness of the PDMS + insulation/adhesion promoter encapsulation in protecting the conductors from corrosive attack by the saline environment. The overall stability of the resistance values across all groups indicates that the encapsulation successfully isolated the platinum traces, preventing significant oxidative degradation or material loss that would typically manifest as a substantial increase in resistance.

Lower resistance with the Overglaze samples is explained by the higher cross-section (thickness of ∼20 μm) and different material (PtAu alloy instead of pure Pt). Even though the technological difference between these samples and the groups consisting of thin-film platinum (thickness of 500 nm) stands, the trend in DC resistance change can be used for stability comparisons. Absolute values favor the manufacturing of ceramic bridging elements by screen printing for their lower electrical resistance.

## 5 Conclusion

Mechanical stability testing of the different layer configurations confirmed the positive benefit of using adhesion promoters on thin-film platinum, with Si_*x*_N_*y*_ enabling adhesion close to reference values on Al_2_O_3_. Overall linear decrease in adhesion for all samples could be observed over the course of 70 days of accelerated aging testing. Though, different materials show different degradation rates. Cross-over points in adhesion should be considered during the selection of the adhesion promoter depending on the envisioned implantation duration. Electrical insulation and DC-resistance measurements further revealed SiO_2_ being the weakest adhesion promoter/insulation layer, thus its usage should be avoided if one aims for long-term stability and reliability of the encapsulation. To further allow the comparison of the thin-film samples to the thick-film samples, more data must be assessed concerning the adhesion capabilities. Measurements for electrical insulation properties are ongoing.

## Data Availability

The datasets presented in this study can be found in online repositories. The names of the repository/repositories and accession number(s) can be found at: FreiData https://doi.org/10.60493/ct9xg-hc715.
